# Life Insurance

**Published:** 1874-11

**Authors:** 


### LIFE INSURANCE.

WE shall gladly commence the discussion of this topic in our paper, and hope to elucidate some facts concerning it, which are not ordinarily presented to the public. The sources of information open to the people on this important subject, are the companies themselves; and their testimony is as erroneous as that of most prejudiced witnesses. We are confident that great frauds are practised upon the public by this system, and we deem it our duty to investigate matters for the benefit of those who look to us for instruction on all important topics. In this connection we ask our readers to supply us with such facts as have come to their knowledge on the subject. Let us have an account of their own experience.

